# Chaperone activity of serine protease HtrA of *Helicobacter pylori* as a crucial survival factor under stress conditions

**DOI:** 10.1186/s12964-019-0481-9

**Published:** 2019-12-03

**Authors:** Urszula Zarzecka, Aileen Harrer, Anna Zawilak-Pawlik, Joanna Skorko-Glonek, Steffen Backert

**Affiliations:** 1 Division of Microbiology, Department of Biology, Friedrich-Alexander-University Erlangen-Nürnberg, Erlangen, Germany; 2 Department of General and Medical Biochemistry, Faculty of Biology, University of Gdańsk, Gdańsk, Poland; 3 Department of Microbiology, Hirszfeld Institute of Immunology and Experimental Therapy, Polish Academy of Sciences, Wroclaw, Poland

**Keywords:** HtrA, Chaperone, Protease, *Helicobacter pylori*, E- cadherin, Virulence factor, Protein quality control system, Stress endurance, Secreted proteins

## Abstract

**Background:**

Serine protease HtrA exhibits both proteolytic and chaperone activities, which are involved in cellular protein quality control. Moreover, HtrA is an important virulence factor in many pathogens including *Helicobacter pylori*, for which the crucial stage of infection is the cleavage of E-cadherin and other cell-to-cell junction proteins.

**Methods:**

The in vitro study of *H. pylori* HtrA (HtrA_*Hp*_) chaperone activity was carried out using light scattering assays and investigation of lysozyme protein aggregates. We produced *H. pylori* ∆*htrA* deletion and HtrA_*Hp*_ point mutants without proteolytic activity in strain N6 and investigated the survival of the bacteria under thermal, osmotic, acidic and general stress conditions as well as the presence of puromycin or metronidazole using serial dilution tests and disk diffusion method. The levels of cellular and secreted proteins were examined using biochemical fraction and Western blotting. We also studied the proteolytic activity of secreted HtrA_*Hp*_ using zymography and the enzymatic digestion of β-casein. Finally, the consequences of E-cadherin cleavage were determined by immunofluorescence microscopy.

**Results:**

We demonstrate that HtrA_*Hp*_ displays chaperone activity that inhibits the aggregation of lysozyme and is stable under various pH and temperature conditions. Next, we could show that N6 expressing only HtrA chaperone activity grow well under thermal, pH and osmotic stress conditions, and in the presence of puromycin or metronidazole. In contrast, in the absence of the entire *htrA* gene the bacterium was more sensitive to a number of stresses. Analysing the level of cellular and secreted proteins, we noted that *H. pylori* lacking the proteolytic activity of HtrA display reduced levels of secreted HtrA. Moreover, we compared the amounts of secreted HtrA from several clinical *H. pylori* strains and digestion of β-casein. We also demonstrated a significant effect of the HtrA_*Hp*_ variants during infection of human epithelial cells and for E-cadherin cleavage.

**Conclusion:**

Here we identified the chaperone activity of the HtrA_*Hp*_ protein and have proven that this activity is important and sufficient for the survival of *H. pylori* under multiple stress conditions. We also pinpointed the importance of HtrA_*Hp*_ chaperone activity for E- cadherin degradation and therefore for the virulence of this eminent pathogen.

## Background

*Helicobacter pylori* is a helical-shaped Gram-negative bacterium, that infects more than half of the human world population [1]. Colonization of the gastroduodenal mucosa by *H. pylori* can lead to chronic gastritis and eventually to the development of peptic ulcers, and represents an important risk factor for malignant alterations such as gastric cancer [2]. To establish a persistent infection, the bacteria must withstand a variety of stressful conditions in the hostile environment of the human stomach. These include heat shock, oxidative, osmotic and acidic stresses, as well as treatment with pharmaceuticals. To persist in this particular niche, *H. pylori* have developed sophisticated stress response systems that allow survival and propagation of the bacteria. *H. pylori* can survive the transient exposure to extreme acid conditions before adherence and growth on the gastric epithelium [3]. For this purpose, *H. pylori* express a specialized urease enzyme locally buffering the pH, which is crucial for survival and adaptation of the bacteria in a changing environment [3]. The pH of the gastric lumen in humans is variable and can reach even pH ~ 1, but the pH near the gastric surface is significantly higher and may be close to neutral [4]. For Gram-negative organisms, including *H. pylori*, the pH in the periplasm is most crucial for bacterial survival and growth. Under in vitro conditions in the laboratory, *H. pylori* propagate best at neutral pH, but under acidic situations, they increase their periplasmic pH using a specialized mechanism, so that the bacterium can be regarded as acid-tolerant neutralophile [4].

*H. pylori* effectively secretes proteins into the extracellular environment, which can be involved in various pathogen-host interactions. This secretome has been studied by mass spectrometry and other techniques, and comprises up to about 125 reported proteins, most notably VacA [5], UreB [6], GGT [7], NapA [8], GroEL [9] and serine protease HtrA (high temperature requirement A) [10]. The proper transport of these proteins across the two bacterial membranes is therefore crucial for the bacteria and requires specific control mechanisms. For example, bacteria subjected to adverse environmental conditions may accumulate damaged proteins. Misfolded polypeptides induce the so-called protein quality control system, comprising chaperones and proteases, whose task is to refold proteins or remove them from a cell by degradation. Depending on the mechanism of action, chaperones can be divided into two major categories, folder and holder chaperones. The folder chaperones (e.g. DnaK and GroEL) are ATP-dependent and participate in the folding and activation of proteins inactivated by certain stress factor, restoring their functionality to the correct conformation and biological properties [11, 12]. On the other hand, holder chaperones (e.g. Ibp and ClpB) can bind to misfolded proteins (independent of ATP), form stable complexes with them and prevent their aggregation; however, this kind of chaperone cannot actively restore the native protein conformation [13, 14]. All bacterial proteins are initially synthesized in the cytoplasm; however, a certain fraction is destined for export to the cellular envelope or extracellular space. Transport of the secreted proteins to the secretory systems and their proper folding at the final destination also requires assistance of chaperone proteins [15, 16]. The bacterial periplasm is particularly predisposed to damage by external factors due to the low selectivity of the outer membrane. Therefore, the extra-cytoplasmic protein quality control system is important for a bacterial cell to ensure proper protein folding and survive stress. The key components of this system are members of the HtrA family of serine proteases. It has been demonstrated that the presence of functional HtrA proteins is necessary for various bacterial species to withstand stressful conditions, such as heat shock or oxidative stress [17–19]. Several known bacterial HtrA homologs exhibit an additional chaperone-like activity, important for the housekeeping roles of these proteins. For example, the presence of the proteolytically inactive *Escherichia coli* HtrA HtrA_*Ec*_ (DegP_*Ec*_) variant (S210A, but retaining its chaperone function) is sufficient to protect *E. coli* cells from accumulation of toxic protein aggregates under heat shock conditions [20].

It has been demonstrated that HtrA is important for survival in the host and/or virulence of several bacterial pathogens such as *Salmonella enterica* serovar Typhimurium [21], *Listeria monocytogenes* [22]*, Klebsiella pneumoniae* [23] and *Yersinia enterocolitica* [24]*.* In *H. pylori,* the presence of functional HtrA_*Hp*_ is necessary to cross the epithelial barrier upon infection. In particular, it cleaves components of the epithelial intercellular junctions, like E-cadherin [25], claudin-8 and occludin [26]. Our very recent publications describe the importance of HtrA_*Hp*_ in response of the cells to stress and biochemical properties of HtrA_*Hp*_ protein [27, 28]. In brief, HtrA_*Hp*_ is important for the survival of *H. pylori* in case of exposure to certain types of stress, for example, heat shock (41 °C), treatment with puromycin, acidic or basic pH (pH 5.2 and pH 7.7) and ionic osmotic stressors (e.g. NaCl) at elevated temperature (39 °C) [27]. Interestingly, the protease function of HtrA_*Hp*_ is preserved over a wide range of pH values [27, 29].

The chaperone activity of the HtrA_*Hp*_ protein is less well understood compared to its proteolytic activity [30]. Due to lack of ATP in the periplasm, HtrAs function presumably as holdases. However, there is growing evidence that the chaperone-like activity of HtrA proteins is involved in the folding and transport of the extra-cytoplasmic proteins, including several virulence factors. For example, secretion of filamentous hemagglutinin (FHA) in *Bordetella pertussis* is HtrA-dependent [31]. The chaperone-like activity of HtrA plays also important roles in the infection process of *Campylobacter jejuni*, a bacterial species closely related to *H. pylori* [32, 33]. The interactions between *C. jejuni* and host cells depend on the chaperone activity of HtrA and bacterial binding to epithelial cells was 5–10 times reduced in the absence of HtrA [32]. The chaperone activity of HtrA alone is sufficient for the growth of *C. jejuni* at high temperature. However, when the temperature and oxygen stresses are combined, the chaperone activity is not sufficient anymore to ensure the survival of the bacterium*.* This observation suggests that severe stress increases the content of unfolded proteins to a point at which chaperone activity is not sufficient to prevent the accumulation of toxic protein aggregates and proteolytic activity is needed to remove the damaged proteins [34].

In the present work, we examined the importance of the HtrA chaperone for the growth of *H. pylori* under stressful conditions and with regard to the virulence of this bacterium*.* For a long time, studies on the chaperone activity of HtrA_*Hp*_ at the cellular level were hampered by a lack of appropriate *H. pylori* Δ*htrA* mutant strains. Our recent report on the construction of a mutant carrying the *htrA*S221A gene coding for the proteolytically inactive HtrA variant in *H. pylori* strain N6 has opened new research possibilities [28]. Using this strain, we demonstrate here that the chaperone activity of the HtrA_*Hp*_ protein plays important roles in the survival of bacteria under severe stress conditions, as well as its role in pathogenesis. We also characterized the chaperone-like activity of this protein in vitro and showed that HtrA_*Hp*_S221A efficiently prevents aggregation of a chemically denatured model substrate, lysozyme. To our knowledge, this is the first report characterizing the chaperone activity of HtrA_*Hp*_ both in vivo and in vitro.

## Methods

### Plasmids, bacterial strains, proteins expression and purification

The strains and plasmids used in this study are listed in Table 1. All HtrA_*Hp*_ proteins were expressed and purified exactly as described previously [27]. In brief, *E. coli* BL21(DE3) transformed with the appropriate plasmid was used to overproduce the proteolytically inactive HtrA_*Hp*_S221A and HtrA_*Ec*_S210A variants (His_6_-tagged), respectively. Bacteria were grown at 37 °C in Luria-Bertani (LB) broth supplemented with 50 μg/mL kanamycin or 50 μg/mL ampicillin to an optical density at 600 nm (OD_600_) 0.6–0.7. Next, 0.5 mM isopropyl-β-D-thiogalactopyranosid (IPTG) was added to induce HtrA protein expression. After lysis and clearing of the lysates by centrifugation, HtrA proteins were purified through nickel-affinity chromatography.

**Table 1:** Bacterial strains and plasmids

Strain/ plasmid	Genotype	Reference/ source
DH5α	*sup*E44 ∆*lac*U169 (φ80 *lac*Z∆M1) *hsd*R17 *end*A1 *gyr*A96 thi-1 *rel*A1	[35]
*E. coli* BL21DE3	F^−^ *ompT hsdS*_*B*_ *(r*_*B*_^*–*^*m*_*B*_^*−*^*) gal dcm*	Novagen
*H. pylori* 26695	Wild-type strain	[36]
*H. pylori* J99	Wild-type strain	[37]
*H. pylori* G27	Wild-type strain	[38]
*H. pylori* 7.13	Wild-type strain	[39]
*H. pylori* N6	Wild-type strain	[40]
*H. pylori* N6∆*htrA*	*H. pylori* N6 *secA*R837K ∆*htrA,* Kan^R^	[28]
*H. pylori* N6 ∆*htrA/htrA*_N6_	*H. pylori* N6 *secA*R837K *∆htrA*/*htrA*_N6_, Cm^R^	[28]
*H. pylori* N6 ∆*htrA/htrA*_N6_ S221A (S/A)	*H. pylori* N6 *secA*R837K *∆htrA*/*htrA*_N6_ S221A, Cm^R^	[28]
pJS17	pQE60, *htrA S210*A from *E.coli* with C-terminal His_6_-tag, Amp^R^	[41]
pUZ3	pET26b, *htrAS221A* from the *H. pylori* 26695 strain with C- terminal His_6_-tag, Kan^R^	[27]

### Bacterial growth conditions

The *H. pylori* wild-type (wt) strains and the derivatives were grown on GC agar (Oxoid) supplemented with 10% donor horse serum (Biowest, France), protease peptone (Oxoid, Germany), 1% vitamin mix, 10 μg/mL vancomycin, 5 μg/mL trimethoprim, 8 μg/mL amphotericin and 10 μg/mL colistin. In the case of mutant *H. pylori*, the media were supplemented with 10 μg/mL kanamycin or 8 μg/mL chloramphenicol, respectively. Bacteria were incubated for 2 days at 37 °C in a 2.5 L anaerobic jar under microaerobic conditions generated by a CampyGen™ sachet (Oxoid, Germany). Colonies were harvested and suspended in BHI (Oxoid, Germany) or Brucella Broth (BB) medium (Sigma-Aldrich, Germany) medium. The bacteria were quantified by OD_600_ measurement and were normalized to 0.35. To induce stress, serial dilutions of a bacterial suspension (5 μL) were spotted onto GC agar plates containing a stress-inducing agent and grown for 6 days. The culture conditions and concentration of stress agents have been described previously [27]. Briefly, we used puromycin (2.5 μg/mL), metronidazole (30 μg/mL) and various pH of the growth medium. The final pH after supplementation and sterilization of the medium was verified using the pH 5 food tester kit (Roth, Germany) and pH was determined as 5.2, 5.6 and 7.7. Standard GC agar plates were prepared at pH 7.1.

The non-ionic (175 mM sucrose) and ionic (85 mM NaCl or 32 mM MgCl_2_) osmolytes were added to the GC medium to induce osmotic shock. The heat shock was induced by increasing the temperature to 39 or 41 °C, respectively. To assess susceptibility to oxidative stress, the disk diffusion assay was performed. For this purpose, 300 μL aliquots of bacterial suspensions in BHI medium (OD_600_ 0.4) were spread over the entire surface of the GC agar plates to generate a lawn. Next, sterile 7 mm diameter disks (Whatman 3 mm) were placed on the top of the agar and 5 μL portions of 2% cumene hydroperoxide or 10-μL portions of 30% hydrogen peroxide were added to induce oxidative stress. The plates were incubated at 37 °C or 39 °C, and after 3 days the inhibition zones were measured. All experiments were performed at least three times.

### In vitro analysis of the chaperone–like activity in HtrA

#### Light scattering assays

**Fig. 1:**
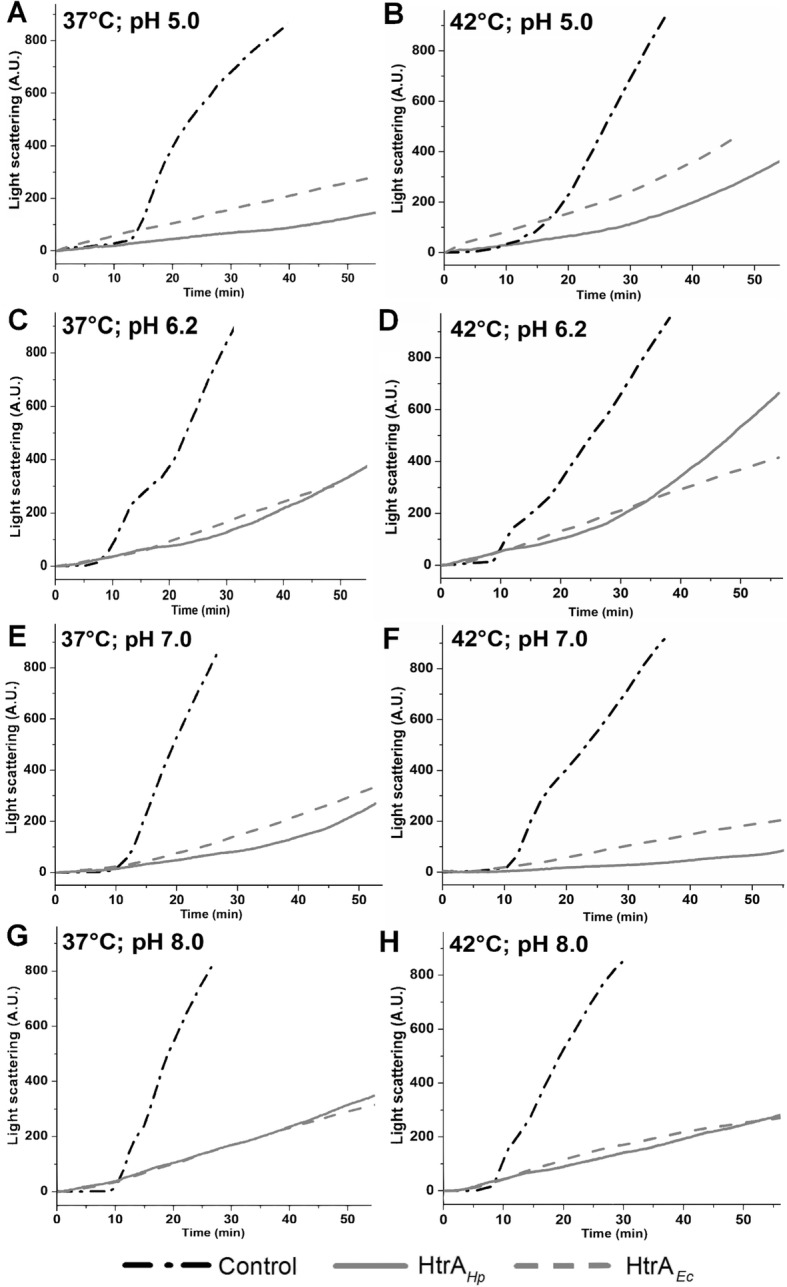
Effect of HtrA_*Ec*_S210A and HtrA_*Hp*_S221A on aggregation of lysozyme at various temperature and pH. The assays were performed in a spectrofluorimeter in thermostated cuvette holders at temperature and pH values indicated in each panel. Samples without HtrA were used as a control. The samples were prepared and analysed as described in Materials and methods. The TCEP concentration used in the assays were: 37 °C (**a**) pH 5.0- 15 mM (**c**) pH 6.2–0.35 mM (**e**) pH 7.0–0.1 mM (**g**) pH 8.0–0.03 mM; 42 °C (**b**) pH 5.0–3.5 mM (**d**) pH 6.2–0.4 mM (**f**) pH 7.0–0.025 mM (**h**) pH 8.0–0.02 mM

The assays were performed exactly as described [42]. In brief, measurements were performed using Perkin- Elmer LS55 Luminescence Spectrometer. Reaction mixtures (900 μL) containing 20 μM lysozyme, 10 μM HtrA S221A (HtrA S/A, inactive variant), 400 mM NaCl, 50 mM HEPES pH 6.2/ 7.0/ 8.0 or 50 mM acetate pH 5.0 were pre-incubated at various temperatures in quartz cuvettes in a thermostated cell holder to obtain proper baselines. Next, the reductant, Tris (2-carboxyethyl) phosphine (TCEP), was added to final concentrations of 20 μM – 15 mM (specified in the legend of Fig. 1) and scattering was measured.

#### Quantification of the lysozyme aggregates

**Fig. 2:**
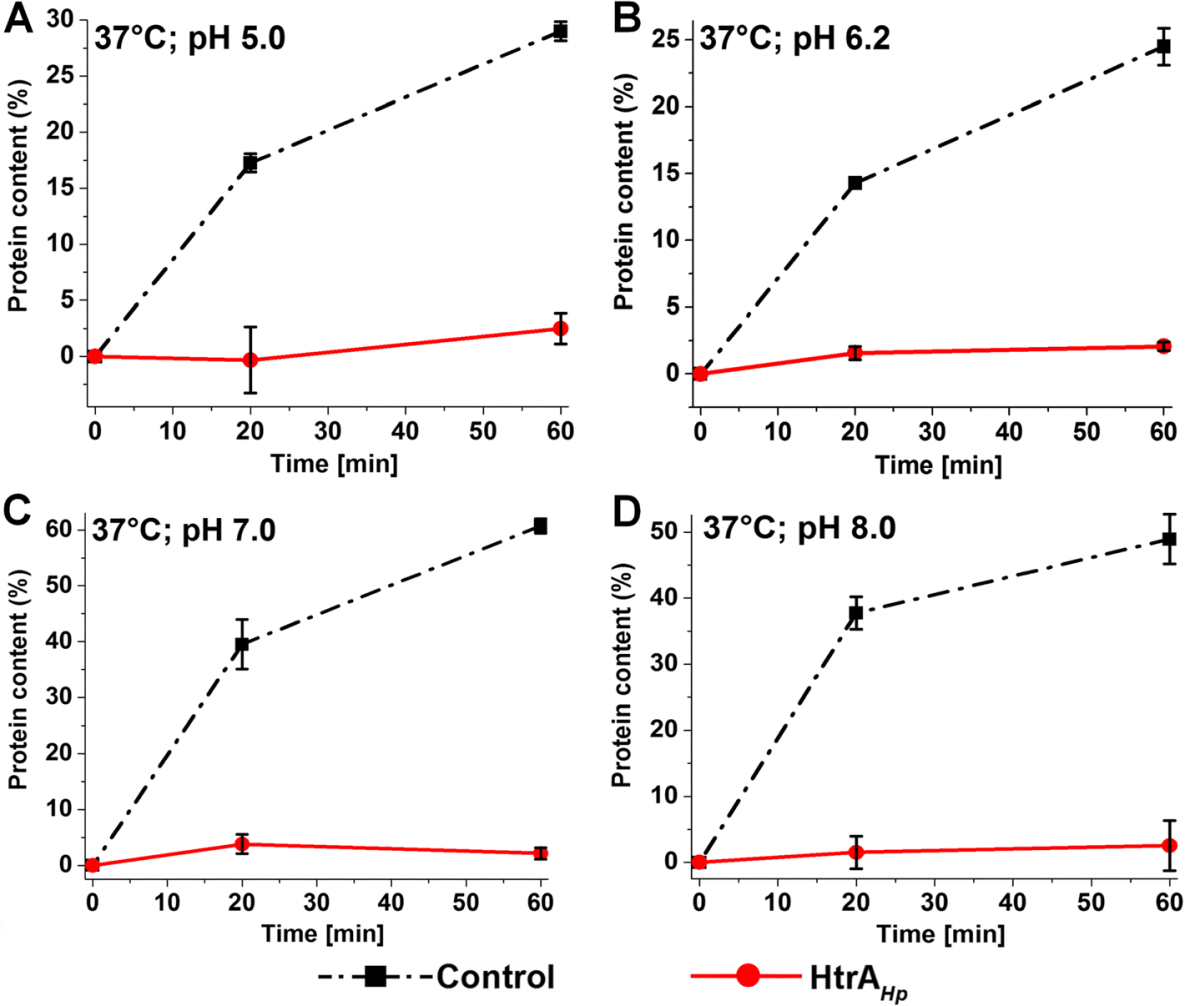
Influence of HtrA_*Hp*_S221A (26695 strain) on the formation of large aggregates of denatured lysozyme. The graphs show the rates of formation of large lysozyme aggregates at 37 °C and pH 5.0 (**a**), 6.2 (**b**), 7.0 (**c**) and 8.0 (**d**). Samples without HtrA were used as a control. The molar ratio HtrA/lysozyme in the samples was 1:2. TCEP concentrations used: **a** 46 mM; **b** 40 mM; **c** 1.4 mM and **d** 0.05 mM. The error bars represent the standard deviation values from three independent measurements

Assays were performed exactly as described [42]. In brief, reaction mixtures (0.4 mL) containing 40 μM lysozyme, 20 μM HtrA S/A, 400 mM NaCl, 50 mM HEPES pH 6.2/ 7.0/ 8.0 or 50 mM acetate pH 5.0 and appropriate concentrations of TCEP (specified in the legend of Fig. 2) were incubated at a temperature specified in the text. Samples (100 μL) were analyzed at three-time points (0, 20 and 60 min). The mock control sample did not contain HtrA_*Hp*_ S/A. The samples were immediately centrifuged at 14,000 x g and 4 °C for 10 min. Pellets were dissolved in 20 μL of 8 M urea. The concentration of proteins was estimated by staining with Amino Black and spectrophotometric measurement as described [43]. As a standard, pure lysozyme with specified concentration was used.

### MKN-28 cell culture, *H. pylori* infection and immunofluorescence staining

MKN-28 cells (JCRB, #0235) were originally isolated from human gastric adenocarcinoma and represent an established polarized cell model to study the gastrointestinal barrier [44, 45]. Cells were cultured in 12-well plates with RPMI1640 medium, containing 4 mM glutamine (Invitrogen, Karlsruhe/Germany), and 10% FCS (Invitrogen, Karlsruhe/Germany) like was described [46, 47]. *H. pylori* wt strains and N6 derivatives were grown on GC agar for 2 days as described above. Next, bacteria were suspended in BB medium and OD_600 nm_ was measured. Infections were performed at a multiplicity of infection (MOI) of 100. After 6 h of infection, the cells were fixed and subjected to immunofluorescence staining as described [47]. Briefly, cell samples were fixed using methanol for 10 min at − 20 °C, followed by permeabilization with 0.5% Triton- X100 for 1 min and blocking with buffer A (1% BSA, 0.1% Tween-20 in PBS) for 30 min. Samples were incubated with mouse α-E-cadherin (CD324, BD Biosciences, San Jose, CA/United States) and rabbit α-*H. pylori* antibodies (Dako, Glostrup/Denmark) for 1 h. As secondary antibodies, FITC (fluorescein isothiocyanate)-conjugated goat anti-mouse (Thermo Fisher Scientific, Darmstadt/Germany), and Alexa Fluor 546-conjugated goat anti-rabbit (Thermo Fisher Scientific, Darmstadt/Germany) were used. Samples were analyzed using a Leica DMI4000B fluorescence microscope and different lasers (Leica Microsystems, Wetzlar/Germany). Images were obtained via LAS AF computer software (Leica Microsystems) that were optimized in brightness and contrast with ImageJ-win64 software (version 1.52n) [48].

### Casein zymography

Bacterial pellets were harvested from GC agar plates and suspended in phosphate buffer saline (PBS). In addition, supernatants obtained from *H. pylori* liquid culture (BB medium supplemented with 0.2% β-cyclodextrin and cholesterol) were used in the experiment. Next, both kinds of samples were mixed with Laemmli buffer (30 mM Tris-HCl, pH 6.8, 5% glycerol, 1.5% sodium dodecyl sulfate, 0.005% bromophenol blue) and loaded onto 10% SDS-PAGE gels containing 0.1% casein (Carl Roth, Germany) and electrophoresed under non-reducing conditions. In the next step, in-gel proteins were renatured by incubation of the gel in 2.5% Triton X-100 solution at room temperature for 60 min with gentle agitation and equilibrated overnight in the developing buffer (50 mM Tris-HCl, pH 7.4, 200 mM NaCl, 5 mM CaCl_2_, 0.02% Brij35) at 37 °C [33, 46, 49]. Transparent bands of proteins with caseinolytic activity were visualized by staining with 0.5% Coomassie Blue R250 as described [50].

### SDS-PAGE and immunoblotting

The SDS-PAGE buffer was mixed with bacterial pellet or supernatant and boiled for 10 min. Samples were separated by SDS-PAGE using 8, 10, 12 or 15% gels. The separated proteins were stained by Coomassie Brilliant Blue (Merck) or blotted onto PVDF membrane (Carl Roth) and blocked with 5% skim milk in TBS-T buffer (200 mM Tris pH 7.4, 1.4 M sodium chloride and 1% Tween-20) for 30 min at room temperature or overnight at 4 °C. The primary antibodies were incubated with membranes for 1.5 h, followed by addition of the secondary antibody for 1 h. Antibody detection was performed as described [46].

### Antibodies

The following antibodies were used: rabbit polyclonal α-HtrA [28]; rabbit polyclonal α-CagA antibody (Austral Biologicals, cat. 5003–9); rabbit polyclonal α-HP1021 [51]; rabbit α-GGT antibody [52]; rabbit α-NapA antibody [52]; rabbit α-UreB antibody (α- HDYTIYGEELK); rabbit α-Lon_*E. coli*_ antibody (Sino Biological, cat. 40,219-RP01); rabbit α-DnaJ_*E. coli*_ antibody (Enzo Life Sciences, cat. ADI-SPA-410); rabbit α-ClpB_*E. coli*_ antibody [53]; rabbit α-GroEL_*E. coli*_ antibody [54]. As a secondary antibody we used goat anti-rabbit polyvalent, horseradish peroxidase-conjugated IgGs (catalogue number #31462, Life Technologies, Darmstadt/Germany).

### HtrA_*Hp*_ secretion assay

The wild-type N6 *H. pylori* and derivatives were suspended in BB medium supplemented with cholesterol and 1% vitamin mix, 10 μg/ml vancomycin, 5 μg/ml trimethoprim, 8 μg/ml amphotericin and 10 μg/ml colistin. The OD_600_ was measured and adjusted to 0.3, followed by *H. pylori* growth for 16-18 h under shaking at 160 rpm and physiological temperature (37 °C). The bacterial pellets and supernatants were prepared by centrifugation at 5000x g for 10 min. Next, Laemmli lysis buffer was added to the pellets. The supernatants were centrifuged at 13,000x g for 30 min (4 °C) and transferred through 0.21 μm sterile filters to remove entire cells and and cell debris, and treated it as described [10]. Bacterial pellets (cellular proteins) and supernatants (secreted proteins) were analysed by Western blotting.

### Protein secretion assay using centrifugal filters

The OD_600_ of wt *H. pylori* strains N6, 26695, J99, G27 and 7.13 as well as N6∆*htrA* mutants was measured and adjusted to 0.2. The bacteria were resuspended in BB medium supplemented with antibiotics as described above and with 1% vitamin mix, cholesterol, and 0.1% β-cyclodextrin and grown for 16 h under shaking at 160 rpm (37 °C). The bacterial pellets and supernatants were prepared by centrifugation at 5000x g for 10 min. The supernatants were centrifuged at 13,000x g for 30 min (4 °C) and transferred through 0.21 μm sterile filters as described above. In the next step, supernatants were concentrated about 18 times by Amicon Ultra 0.5 mL Centrifugal Filters-10 K and total protein concentration of the supernatant was measured by NanoDrop (Thermo Scientific). The concentration of all supernatant samples was adjusted to 80 μg/μL and Laemmli lysis buffer was added. Samples were analysed by Western blot or SDS-PAGE Coomassie Brilliant Blue staining. The relative amounts of HtrA_*Hp*_ secreted by several *H. pylori* strains were measured by densitometry of Western blot band intensities as described below. Next, the same amounts of total proteins from the supernatants were used for cleavage assays with β-casein (8 μg of β-casein per reaction). Reactions were performed in reaction buffer (50 mM HEPES pH 7.4; 100 mM NaCl) for 16 h at 37 °C. The reactions were terminated by the addition of Laemmli lysis buffer and then resolved in 15% SDS- PAGE and analysed using densitometry. Sample without supernatant was used as mock control and this band was set as 100%.

### Quantification of Western blot band intensities and statistics

To quantify band intensities on immunoblots, we performed densitometric measurements using the 1DScan EX program (Scanalytics Inc., United States). The statistics One-Way Anova (Bonferroni test) approach was performed using OriginPro 8.5.1 software (OriginLab Corp., USA). Statistical significance was defined by *p* ≤ *0.05* (*), *p* ≤ *0.01* (**) and *p* ≤ *0.001* (***).

## Results

### HtrA_*Hp*_ exhibits chaperone activity

Functional studies in *E. coli* and other bacteria indicate that proteins of the HtrA family do not have only proteolytic activity, but also chaperone activity [55]. In the present work, we investigated the chaperone capabilities of HtrA_*Hp*_ to inhibit aggregation of unfolded proteins. To this end, we first tested under which conditions HtrA_*Hp*_ exhibits chaperone activity using reduced lysozyme as a substrate. Aggregation of lysozyme was initiated chemically by using a reducing agent, which reduced disulfide bridges stabilizing the protein structure [56]. HtrA_*Ec*_ (DegP_*Ec*_) was used as a positive control, because chaperone activity of this HtrA homolog is best studied and was first published [18, 20]. The process of lysozyme aggregation was monitored by light scattering measurements, a method that enables to visualize formation of aggregated protein particles in a course of time. To study the pH and/or temperature dependence of the HtrA_*Hp*_ chaperone activity, the reactions were performed at pH 5.0, 6.2, 7.0, and 8.0, and temperatures of 37 °C and 42 °C (Fig. 1). Generally, significant suppression of the lysozyme aggregation by HtrA_*Hp*_ was observed under all tested conditions. The control chaperone, HtrA_*Ec*_ (DegP_*Ec*_), reduced lysozyme aggregation to the similar extent. Both proteins were most efficient at neutral pH (7.0). To characterize the efficiency of HtrA_*Hp*_ as a chaperone, we evaluated amounts of the denatured lysozyme precipitates formed in the presence/absence of HtrA_*Hp.*_ We found that co-incubation with HtrA_*Hp*_ drastically reduced the quantity of large aggregates under all tested conditions (Fig. 2). Taken together, we concluded that HtrA_*Hp*_ exhibits a chaperone activity which is effective under a wide range of physiological temperatures and pH values.

### Effect of the HtrA chaperone activity on the viability of *H. pylori* under various stress conditions

**Fig. 3:**
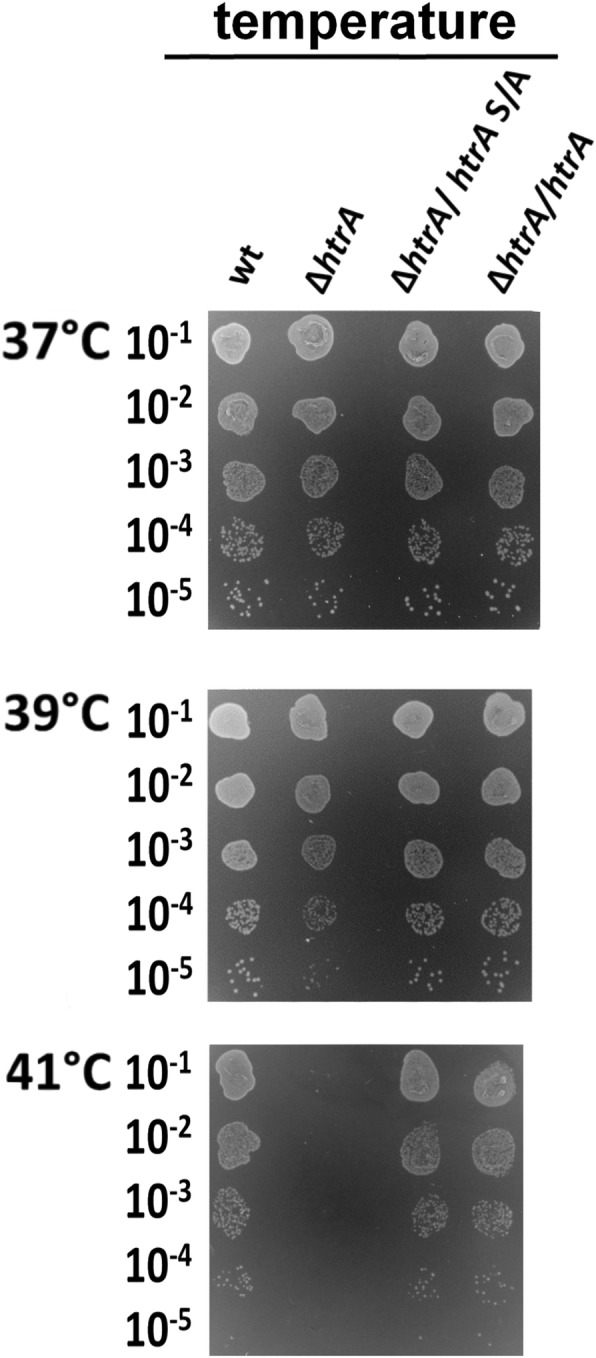
Effects of temperature on the growth of *H. pylori.* Tested strains were: N6 wt, ∆*htrA*, complemented strains with wt *htrA* (∆*htrA*/*htrA*) and with proteolytically inactive variant of *htrA* (∆*htrA*/*htrA*S/A)*.* Serial dilutions of bacterial cultures were spotted onto GC agar plates and bacterial growth at three temperatures was assessed after 6 days of incubation on GC agar plates. The experiments were performed in triplicates and representative examples are shown

We have recently identified that lack of *htrA* significantly affects the survival of *H. pylori* under stressful conditions [27]. In the present study, we aimed to investigate the impact of proteolytically inactive HtrA in *H. pylori*, which will allow us to evaluate the role of chaperone activity of this protein in the bacterium. For these tests, we used *H. pylori* N6 wt and its derivatives N6 ∆*htrA* (∆*htrA),* N6 ∆*htrA/htrA*_N6_ S221A (∆*htrA/htrA* S/A) and the wt complemented strain N6 ∆*htrA/htrA*_N6_ (∆*htrA*/*htrA*) as control (Additional file 1: Figure S1). We observed no visible growth defect for ∆*htrA* and ∆*htrA/htrA* S/A when they were grown under standard conditions (37 °C, GC agar medium) (Fig. 3a and Additional file 1: Figure S2A). As we have determined in recent studies, at 39 °C, ∆*htrA* grew slower and at 41 °C we observed a significant decrease in survival compared to the control strains [27]. To the contrary, ∆*htrA*/*htrA* S/A grew similarly to N6 wt and wt complemented strain ∆*htrA*/*htrA*. Therefore, we assumed that proteolytically inactive HtrAS221A can suppress the temperature sensitive phenotype of the ∆*htrA* strain (Fig. 3 and Additional file 1: Figure S2A).

**Fig. 4:**
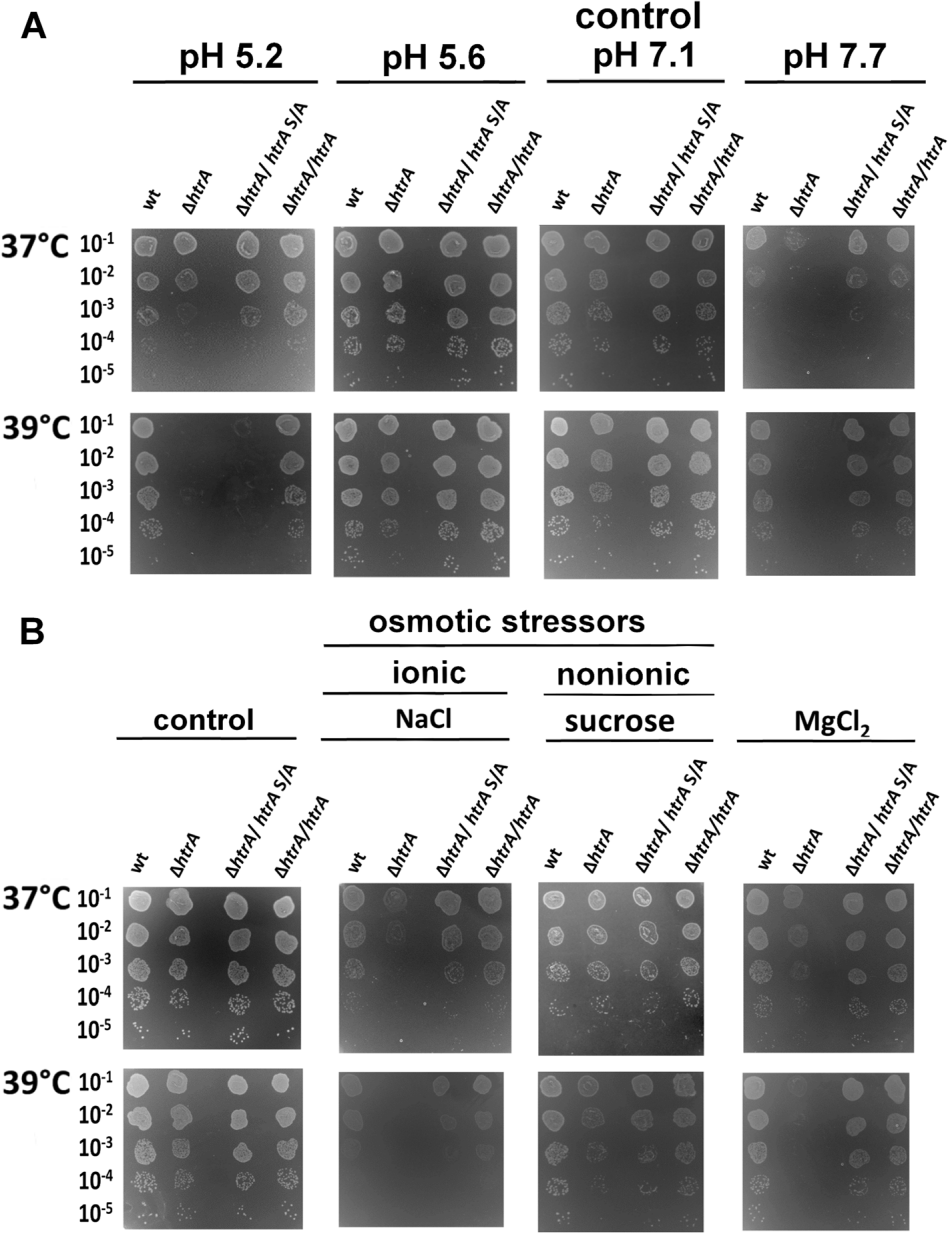
Effects of pH (**a**) and osmotic stress (**b**) on the growth of *H. pylori.* We tested the following strains: N6 wt, ∆*htrA*, complemented strains with wt *htrA* (∆*htrA*/*htrA*) and with proteolytically inactive variant of *htrA* (∆*htrA*/htrAS/A)*.* For osmotic stress, bacteria were grown on GC agar plates for 6 days in the presence of sucrose (175 mM), NaCl (85 mM) or MgCl_2_ (32 mM) at 37 or 39 °C. The experiments were performed in triplicates and representative examples are shown

As next we tested the effects of pH stress. The pH of the standard GC agar medium for *H. pylori* growth is 7.1. The change in pH to 5.2 or 7.7 caused a significant decrease in the survival of ∆*htrA*, especially at elevated temperature (39 °C). Again, the presence of HtrAS221A enabled growth of bacteria under the majority of chosen experimental conditions. The only exception was a combination of pH 5.2 and 39 °C which was lethal for both, ∆*htrA* and ∆*htrA/htrA* S/A (Fig. 4a and Additional file 1: Figure S2B).

**Fig. 5:**
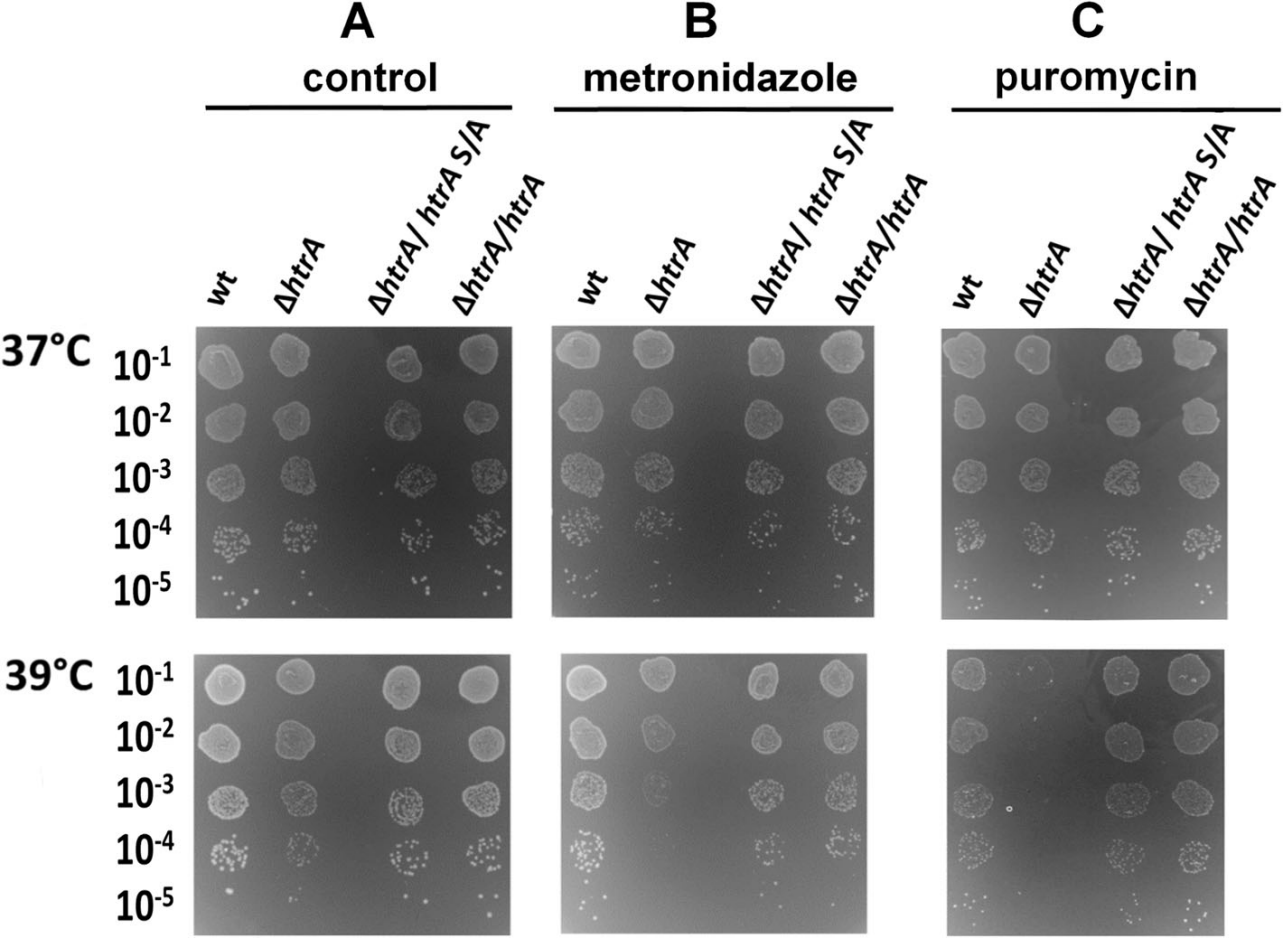
Effects of metronidazole (**b**) and puromycin (**c**) on the growth of *H. pylori.* The following strains were tested: N6 wt, ∆*htrA*, complemented strains with wt *htrA* (∆*htrA*/*htrA*) and with proteolytically inactive variant of *htrA* (∆*htrA*/htrAS/A)*.* Serial dilutions of bacterial cultures were spotted onto GC agar plates without (**a**) and with (**b**) metronidazole or **(c)** puromycin and bacteria growth for 6 days. The experiments were performed in triplicate and the representative examples are shown

In the following set of experiments we tested osmotic stress using ionic and nonionic stressors. We have noted that sucrose (175 mOsm), which is non-ionic osmoticum, did not affect the viability of any of the tested strains (Fig. 4b and Additional file 1: Figure S2C). However, the stress caused by the increased concentration of salts, e.g. NaCl or MgCl_2_, had a significant impact on *H. pylori* growth. Visible colonies were smaller compared to controls, and ∆*htrA* grew very poorly at 37 °C, while at 39 °C no colonies were formed (Fig. 4b and Additional file 1: Figure S2C). Also, under these conditions expression of HtrAS221A was beneficial for *H. pylori*. Although the salt stress sensitivity of ∆*htrA* was not fully suppressed by HtrAS221A in the presence of NaCl at elevated temperature (39 °C), growth of bacteria was weaker. Thus, we can assume that the chaperone activity of HtrAS221A plays important protective role under osmotic stress conditions (Fig. 4b). We have reported that the lack of HtrA did not influence survival of *H. pylori* under oxidative stress induced by H_2_O_2_ or cumene hydroperoxide [27]. Accordingly, a lack of the proteolytic activity of HtrA_*Hp*_ did not affect growth of bacteria in the presence of the oxidants (Additional file 1: Figure S3)*.* However, bacterial survival in the presence metronidazole was supported by the presence of HtrA_*Hp*_ and HtrAS221A_*Hp*_, while the metronidazole-treated ∆*htrA* cells grew poorly at 39 °C (Fig. 5b and Additional file 1: Figure S2D). Metronidazole is commonly used in a therapy against *H. pylori* [57], which is a redox-active prodrug that needs to be activated by reduction of the nitro group attached to the imidazole ring. This reduction step leads to the production of DNA-damaging compounds, which can kill the bacterium [58]. A further action mechanism of metronidazole may lead to production of nitroradical anions that reduce O_2_ and thereby generate reactive oxygen species [59, 60]. Therefore, the contribution of HtrA to protection against oxidative stress is possible.

Finally, we tested the effects of puromycin treatment. This antibiotic compound leads to the premature release of unfinished protein chains from ribosomes [61] and it causes the accumulation of improperly folded proteins [62]. Exposure of *H. pylori* to puromycin retarded the growth of all tested strains at 37 °C. At 39 °C, ∆*htrA* did not form colonies in contrast to ∆*htrA/htrA* S/A; hence, expression of HtrAS221A can suppress the negative effects of puromycin (Fig. 3c and Additional file 1: Figure S2E).

### Lack of *htrA* does not affect the level of secreted proteins

**Fig. 6:**
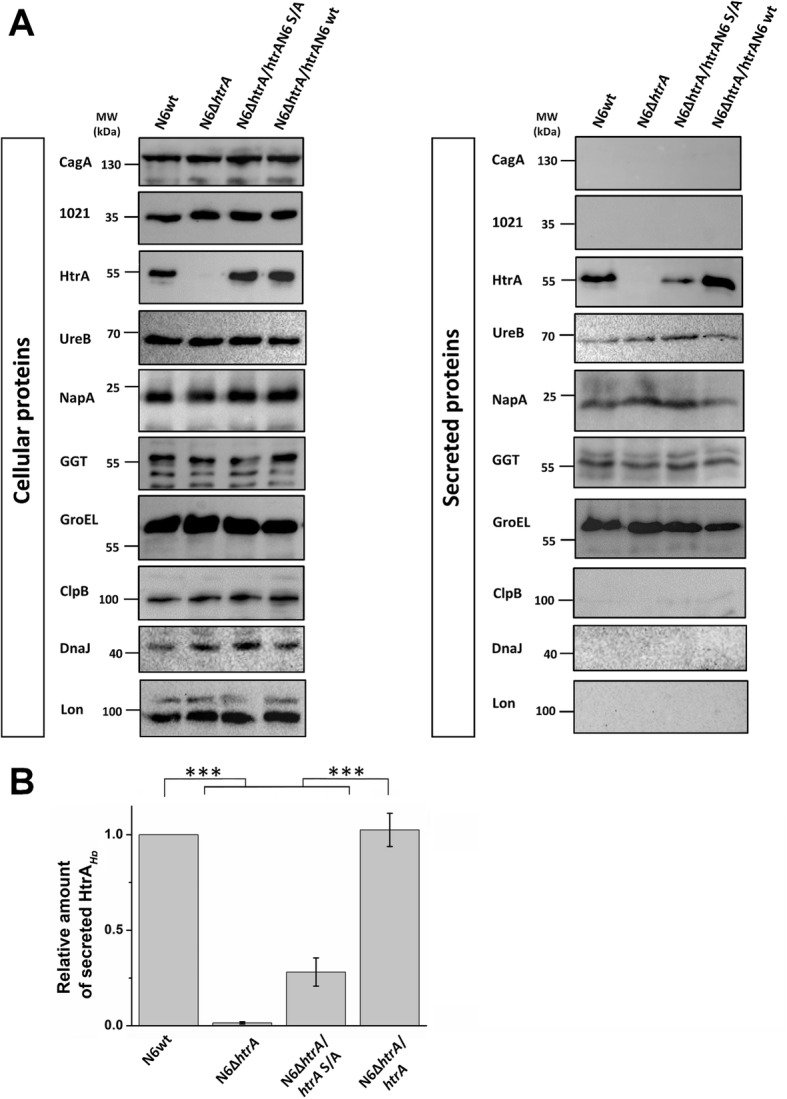
Mutation of HtrA_*Hp*_ has no effect on the secretion of proteins. Proteolytic activity of the HtrA_*Hp*_ protein is important in the process of secretion of this protein outside the bacterial cell. Total proteins were labelled as a pellet, bacteria-free secreted samples was labelled as a supernatant. **a** Western blotting analysis of *H. pylori* lysates. The CagA protein was used as a control (as a non-secreted protein). **b** The band intensities of secreted proteins were quantified densitometrically using 1Dscan Ex program and the relative amount of secreted protein is given. Significant differences were analyzed using Bonferroni test (***p < 0.001). The standard error of mean (SEM) was calculated using at least three repetitions. Immunodetection using Lon antibodies was performed in duplicate

Determination of the physiological function of the HtrA_*Hp*_ protein in the bacterial cell has so far been significantly hampered due to the lack of a mutant lacking the functional *htrA*_*Hp*_ gene. Previous attempts to inactivate the *htrA*_*Hp*_ gene were unsuccessful, despite the usage of more than 100 *H. pylori* strains of various origin [63]. Now we obtained a ∆*htrA* mutant in strain N6, and previously published analyses showed that in selected *H. pylori* strains (N6, 26695, J99, HPAG1, Shi470 and India7) no additional protease was found that could replace HtrA in function [28]. Using the *htrA* mutant strains we undertook an investigation how the lack of HtrA protein or its proteolytic activity in the bacterial cell may affect the level of cellular and secreted proteins. In this experiment, we inspected well-known secreted proteins of *H. pylori* such as, HtrA, UreB, NapA, GGT and GroEL, while intracellular HP1021, ClpB, DnaJ, Lon and CagA proteins served as negative controls. We found that the cellular content of the tested proteins was similar in all tested strains. Western blotting confirmed the lack of the HtrA protein in the corresponding mutant strain. In the case of CagA, HP1021, ClpB, DnaJ and Lon proteins, it was visible that they are not secreted outside the cell as expected. This result also confirmed that there were no broken cells or cell debris in the supernatant. Interestingly, the ∆*htrA/htrA* S/A mutant secreted significant less HtrA protein compared to wt and complemented ∆*htrA*/*htrA* (Fig. 6). Analysis of the level of secreted UreB, NapA, GGT and GroEL proteins, however, indicated no significant differences in the level of these proteins between the tested strains (Fig. 6 and Additional file 1: Figure S4).

### Expression of HtrA by *H. pylori* is important for disruption of E-cadherin in the adherens junctions of polarized MKN-28 cells

**Fig. 7:**
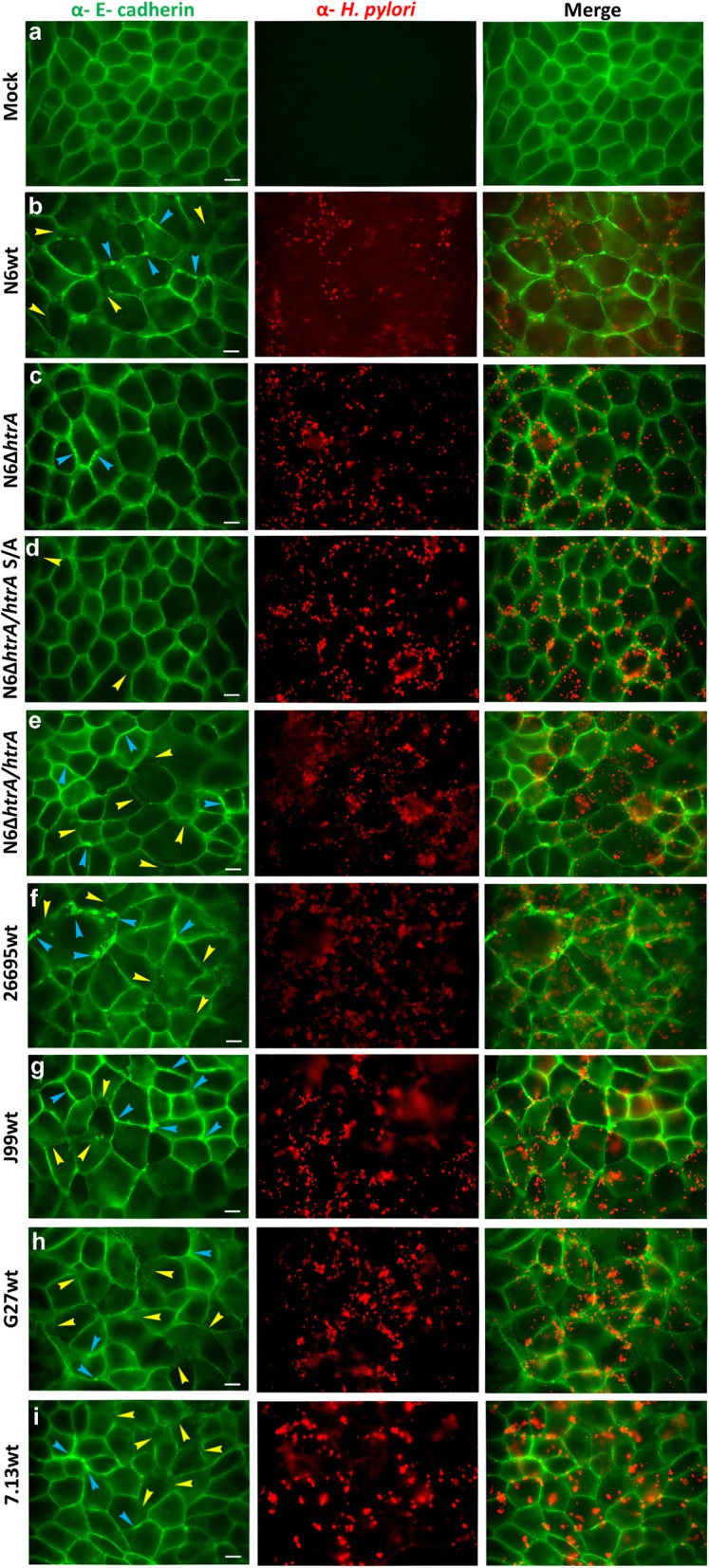
Importance of HtrA on disruption of E- cadherin based cell-to-cell junctions during infection with *H. pylori*. Polarized MKN-28 cells were left untreated (**a**) or infected for 6 h with *H. pylori* N6 wt (**b**)**,** N6∆*htrA* (**c**)***,*** N6∆*htrA/htrA* S/A (**d**), N6∆*htrA/htrA* (**e**), 26695 wt (**f**), J99 wt (**g**), G27 wt (**h**) and 7.13 wt (**i**). The cells were subjected to immunofluorescence staining detected E-cadherin (green) and *H. pylori* (red). Arrowheads mark cells showing significantly downregulated (blue) or disrupted (yellow) E-cadherin signals. The scale bar corresponds to 10 μm

**Fig. 8:**
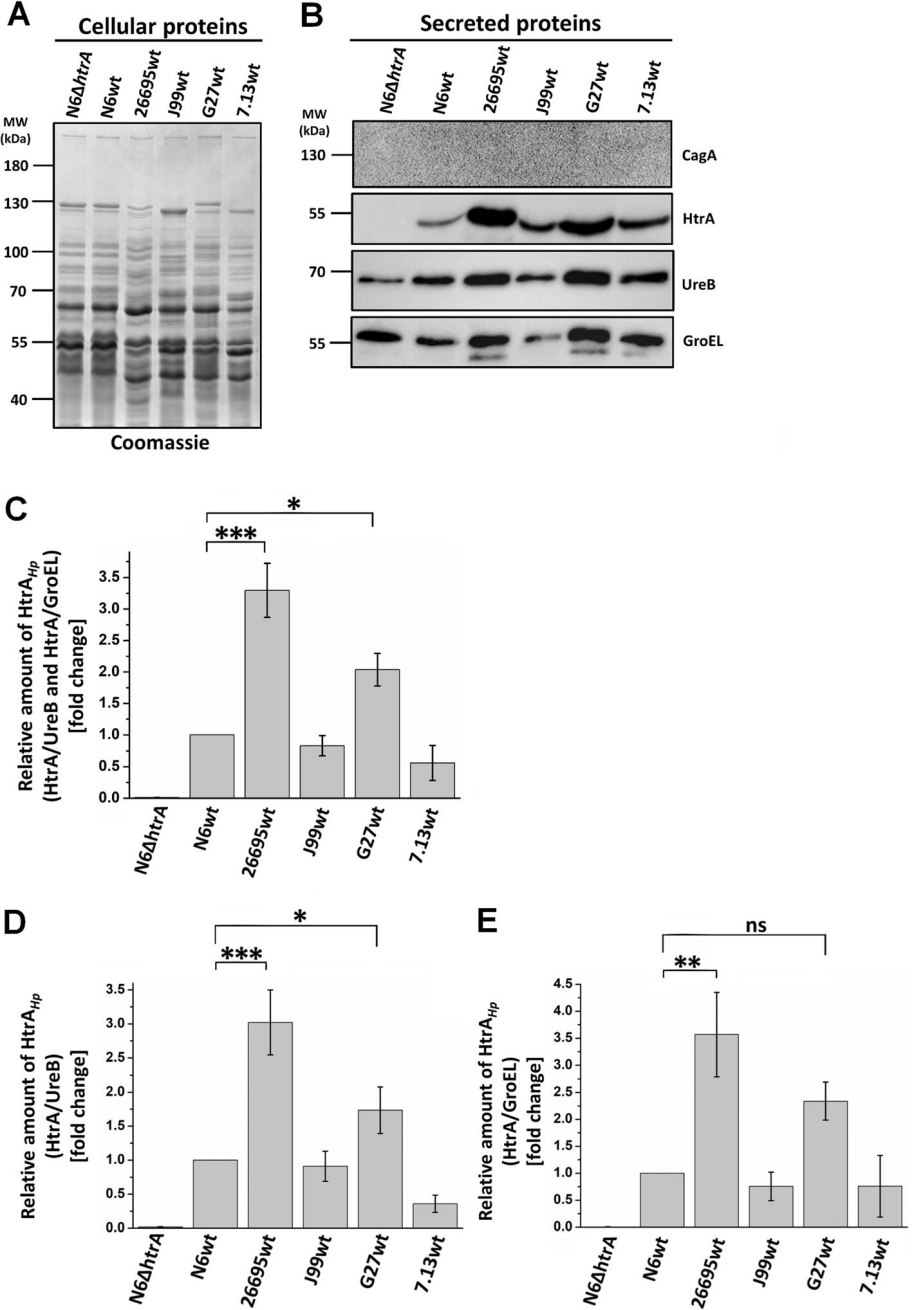
Differences in HtrA secretion by several *H. pylori* strains. **a** Total cellular protein profiling of the *H. pylori* lysates by Coomassie-stained SDS-PAGE. **b** Western blot analysis of level secreted proteins HtrA, UreB and GroEL. CagA was used as a control. **c**-**e** The amount of HtrA relative to other secreted proteins (UreB and GroEL). Significant differences were analyzed using Bonferroni test (*p* < *0.05* *, *p* < *0.01* ** and *p* < *0.001* ***). The standard error of mean (SEM) was calculated using at least three repetitions. ns means no significant differences

**Fig. 9:**
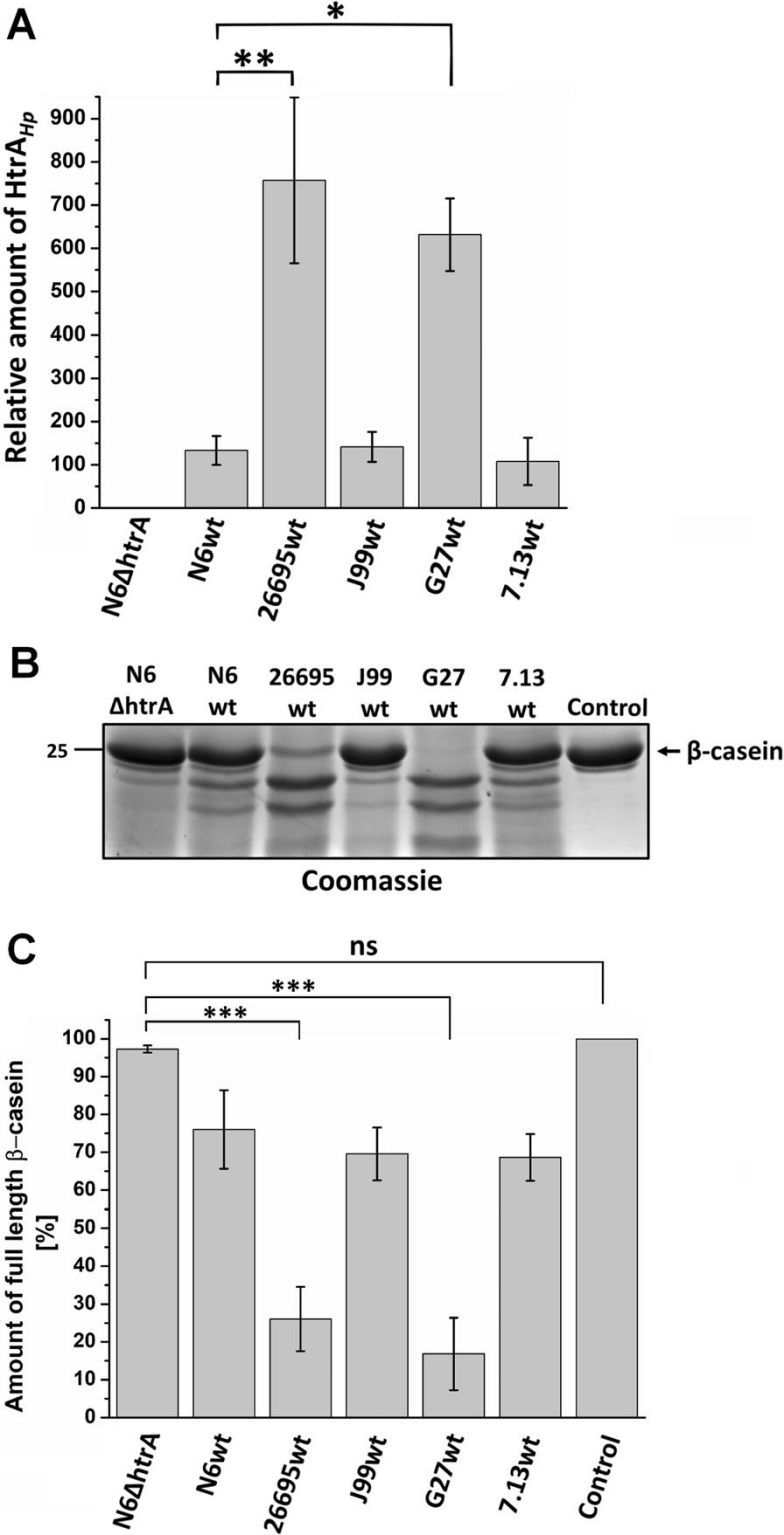
Differences in proteolytic activity of secreted HtrA by several *H. pylori* strains. **a** Relative amount of secreted HtrA_*Hp*_ was measured by Western blotting using densitometric method. **b** Digestion of β-casein using supernatants from overnight grown *H. pylori* strains. The Coomassie-stained gel showed the result after 16 h incubation of reaction mixture. **c** The band intensities of β-casein were quantified densitometrically and sample without supernatant was set as a 100%. Significant differences were analyzed using Bonferroni test (*p* < *0.05* *, *p* < *0.01* ** and *p* < *0.001* ***). The standard error of mean (SEM) was calculated using at least three repetitions. ns means no significant differences

**Fig. 10:**
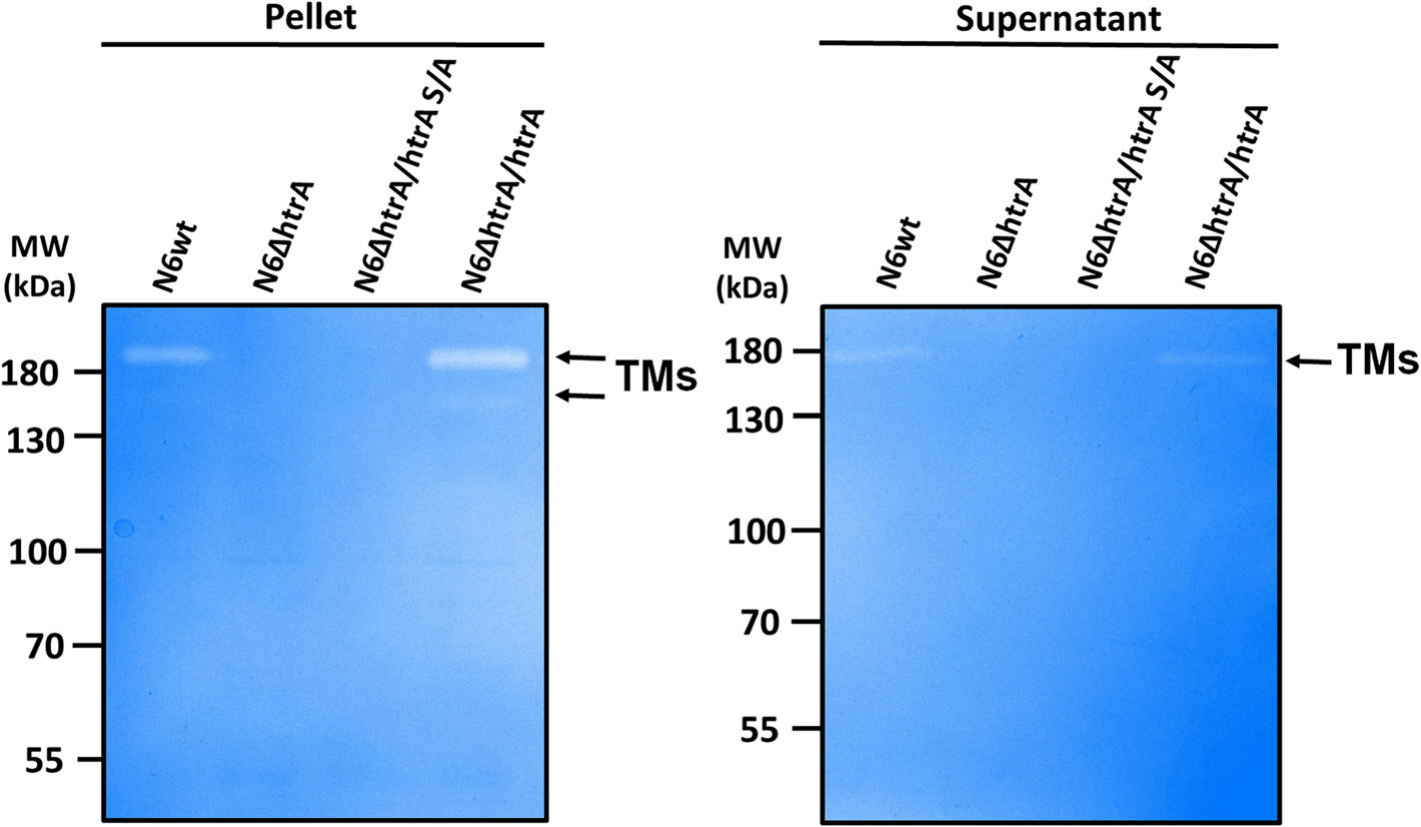
Analysis of HtrA proteolytic activity in *H. pylori* N6 wt and mutant strains. The proteolytic activity of HtrA was analysed by casein zymography. Supernatants and pellets from liquid culture were tested. The position of proteolytically active HtrA trimers (TMs) on the gels is indicated with arrows

In the next experiments we aimed to study the effect of lack of *htrA* during infection in comparison with several wt *H. pylori* strains. For this purpose, confluent polarized MKN-28 epithelial cell monolayers were infected for 6 h with wt strains N6, 26695, J99, G27 and 7.13 as well as ∆*htrA,* ∆*htrA/htrA* S/A and complemented strain ∆*htrA*/*htrA.* Infected and mock control cells were fixed and stained for immunofluorescence visualization using antibodies against the E- cadherin (green) and *H. pylori* (red). The uninfected mock control cells showed the typical E-cadherin signals between the neighboring cells in the monolayer as expected (Fig. 7a). When cells were infected by wt strains or by complemented strains, we observed disruption of E-cadherin in many areas of the cell culture (Fig. 7a, b, e-i, yellow arrows). The E-cadherin signals for some cells were downregulated (blue arrows) and were characterized by mislocalization of the protein. In contrast, infection with the ∆*htrA* and ∆*htrA/htrA* S/A mutant strains resulted in no or only very low levels of disruption and mislocalization of E-cadherin (Fig. 7c, d). Interestingly, we noticed that level of disruption of intercellular junctions differs between strains. Therefore, we compared amounts of HtrA secreted by the examined *H. pylori* strains. For this purpose, we prepared liquid *H. pylori* cultures in BB medium containing 0.2% β-cyclodextrin and cholesterol. Indeed, we observed significant differences in the secretion of the HtrA protein between the tested strains (Fig. 8b and Fig. 9a). For 26695 and G27, the levels of secreted HtrA were the highest and it was even 6–7 times greater compared to N6, J99 and 7.13, which secreted less HtrA. Strain J99 also secreted very low amounts of UreB and GroEL proteins. Only strains 26695 and G27 showed differences in HtrA level in relation to other secretory proteins. Probably these strains are characterized by the increased secretion of numerous of secreted proteins, not only HtrA. To verify proteolytic activity of secreted HtrAs, we incubated equal amounts of *H. pylori* supernatant protein with β-casein, a universal substrate for proteases. We found that the level of digested β-casein correlated with the amount of HtrA secreted by the tested *H. pylori* strains (Fig. 9). In the case of strains 26695 and G27, the majority of the full-length β-casein protein was digested, and for N6, J99 and 7.13 we obtained about 20–30% digested substrate. In the ∆*htrA* mutant, we observed a slight digestion of β-casein, suggesting that other active protease(s) is/are present in the supernatants (Fig. 9b and c). To have additional information about the proteolytic activity of secreted HtrA_*Hp*_, we further tested N6 wt, ∆*htrA,* ∆*htrA/ htrA* S/A and the complemented ∆*htrA*/*htrA* strain using casein zymography. For this purpose, we used samples from liquid culture, pellets and supernatants after concentration. As expected, for ∆*htrA* and ∆*htrA/htrA* S/A, we observed that they are unable to digest casein in contrast to N6 wt and ∆*htrA*/*htrA*, which form visible HtrA trimers (Fig. 10).

## Discussion

For many pathogenic bacteria, HtrA has been characterized as an important virulence factor [64, 65]. In the present study, we focused on characterizing the chaperone activity of HtrA_*Hp*_ in vivo and in vitro that has not yet been investigated. We studied the in vitro chaperone activity in *H. pylori* using two methods, which allowed us to observe the resulting lysozyme aggregates after the use of a reducing factor in the presence or absence of HtrA. We were able to show that HtrA_*Hp*_ efficiently inhibits the lysozyme aggregation process similar to the HtrA_*Ec*_ (DegP_*Ec*_) protein, whose chaperone activity has already been described in our previous reports [20, 42]. Moreover, this activity is effective under different conditions of pH and temperature stresses.

Using recombinant proteins, the chaperone activity was previously described for the HtrAs of *Campylobacter jejuni* [34], *Stenotrophomonas maltophilia* [42], *Haemophilus parasuis* [66] and *Chlamydia trachomatis* [67], which is similar to our present findings for HtrA_*Hp*_ and HtrA_*Ec*_ (DegP_*Ec*_). Interestingly, the chaperone activity was also detected in the human orthologs (HTRA 1–3 [68–71] and suggested for HTRA4 [72]) and a yeast HtrA protein [73]. Thus, conservation of HtrA chaperone activity in the evolution of multiple species may indicate their overall importance, however, this activity is still not fully understood.

Together with our previous publication [27], we described here the role of HtrA (both chaperone and proteolytic activities) in *H. pylori* under stress conditions. For these tests, we used N6 wt and its mutants N6 ∆*htrA* (∆*htrA*) and N6 ∆*htrA/htrA*_N6_ S221A (∆*htrA/htrA* S/A) as well as its complemented strain ∆*htrA/htrA*_N6_ (∆*htrA*/*htrA*), for which construction methods have been presented in previous publications [27, 28]. The strains have been exposed to thermal, osmotic and pH stressors as well as to the antibiotics puromycin and metronidazole. All of these stresses can affect the proper functioning of a bacterial cell. Puromycin causes the premature termination of protein synthesis in the translation process, which results in a large amount of misfolded proteins that can form toxic aggregates for cells [61, 62]. Metronidazole induces the production of DNA damaging radicals [74] and probably other reactive species that denature other cellular macromolecules are also formed [59, 60]. Other tested stresses such as osmotic, temperature, acid, basic and oxidative factors can result in protein denaturation and aggregation, which can lead to cell death. Under such circumstances, HtrA acts as an element of the cellular protein quality control system and can prevent the formation of protein aggregates through their degradation (proteolytic activity) or by keeping them in a soluble state (chaperone activity). In our previous publication, we reported that the *htrA* deletion mutant in *H. pylori* is more sensitive to the majority of stress tested in comparison to the wt strain [27]. Now we demonstrated that the strain lacking proteolytic activity of HtrA showed a high degree of resistance to the stresses studied and the mutant grew similarly compared to the N6 wt strain under the majority of tested experimental conditions. These results allowed us to propose that the HtrA chaperone activity is sufficient for *H. pylori* to withstand a variety of adverse environmental conditions. By comparison, the chaperone activity of *C. jejuni* HtrA also plays important roles in protection of the bacteria against heat or oxidative stress. However, when the bacteria were exposed to both stresses, the strain lacking proteolytic activity did not survive [34].

In several bacterial species, HtrA homologs are involved in protein secretion [75]. To examine a potential role of HtrA for protein secretion by the *H. pylori* cell, we analyzed the presence of typical secreted proteins in the culture supernatant. We investigated the expression and secretion levels of UreB, NapA, GGT, and HtrA proteins. The urease is an important survival and virulence factor, which is produced in large amounts by *H. pylori*. This protein allows the bacteria to colonize and induce inflammatory responses in the gastric epithelium. The enzymatic activity of urease causes hydrolysis of urea into ammonia, which consequently leads to neutralizing the acid environment around the bacteria [76] and urease plays a role in inflammasome activation [77] and induction of hypoxia-induced factor, HIFα [78]. Neutrophil-activating protein (NapA) is important in inducing neutrophil recruitment through the endothelium and can stimulate host cell production of reactive oxygen species [8, 79]. Tests have shown that NapA plays a role in preventing oxidative DNA damage and is crucial for *H. pylori* colonization [8]. On the other hand, GGT is a bacterial virulence factor, which causes glutamine and glutathione consumption as well as the production of ammonia and reactive oxygen species in the host. Moreover, GGT can induce apoptosis and necrosis in gastric epithelial cells [80]. Interestingly, we did not observe differences in the expression and secretion levels of UreB, NapA and GGT in the ∆*htrA* and ∆*htrA/htrA* S/A strains, and also did not observe changes in the survival of the examined strains (Additional file 1: Figure S2). In the case of HtrA_*Hp*_, we saw the lower amount of secreted HtrA in the ∆*htrA/htrA* S/A strain suggesting that the proteolytic activity of HtrA_*Hp*_ is required for efficient secretion into the extracellular environment. This finding is in contrast to previous observations made with *C. jejuni* HtrA, in which the protease activity played no role for HtrA_*Cj*_ secretion [33]. The reason for these differences are yet unknown and should be studied in future experiments.

We also checked the expression and secretion levels of several chaperone proteins such as GroEL, ClpB, DnaJ in the various genetic *htrA* backgrounds of *H. pylori*. The presence of genes encoding these proteins in *H. pylori* has been demonstrated in earlier reports [9, 81, 82], but are not characterized at much detail. However, it is known that in *H. pylori* the levels of GroEL increase during heat shock [83] and GroEL_*Hp*_ plays a role in regulating the activity of urease enzyme [84]. In addition, *H. pylori* without *clpB* are more sensitive to temperature stress [81]. The co-chaperone DnaJ was shown to stimulate ATPase activity of DnaK, enhancing the recognition and binding of DnaK substrates [82]. In our present studies, we did not observe changes in the expression level of chaperones under physiological conditions in vitro. Perhaps, this could change under any stressful conditions. Among the *H. pylori* chaperones, only GroEL is known to be secreted into the extracellular environment. In the case of *C. jejuni*, *htrA* deletion resulted in the overproduction of DnaK and ClpB, while in the corresponding protease-deficient S/A mutant only the overproduction of ClpB was observed in vitro [34]. Interestingly, at elevated temperatures, the overproduction of DnaK and ClpB proteins was clearly visible for both strains [34].

As negative control, we checked the level of CagA, which is probably the best studied *H. pylori* virulence factor. Moreover, translocated CagA can dysregulate signal transduction of gastric epithelial cells, which is involved in chronic inflammation and malignancy by changing cell polarity, apoptosis, and proliferation [63]. It is known, that CagA is an effector protein translocated into host cells and not secreted into the supernatant, so we used it as a control for this experiment, and we observed the lack of CagA in our supernatant samples.

In addition, we have not seen an effect of the lack of *htrA* on the expression of the HP1021 protein that is encoded next to *htrA* and involved in the chromosomal replication process [51] and on the Lon protease, which is associated with specific recognition and degradation of incomplete, damaged, and non-native proteins [85].

To learn more about the role of HtrA in the virulence process of *H. pylori*, we checked the impact of HtrA deficiency and its proteolytic activity during one of the most important stages of infection, it means the disruption of E-cadherin-based junctions between neighboring epithelial cells. As a model system, we infected polarized MKN-28 cells with the above discussed strains followed by immunofluorescence of E-cadherin. Non-infected control cells showed the proper cell-to-cell junctions with E-cadherin staining. Cells infected by N6 wt, N6 complemented ∆*htrA/htrA* or other wt strains (26695, J99, G27 and 7.13) showed the significant disruption of junctions between cells, as indicated by the dispersed E-cadherin signals and changes in the shape of MKN-28 cells (Fig. 7). In contrast, during infection with ∆*htrA* and ∆*htrA/htrA* S/A mutant strains the epithelial cell structure remained widely preserved, and only very low levels of disruption and mislocalization of E-cadherin were observed (Fig. 7a, b, e). These observations are in line with previous results that HtrA_*Hp*_ is able to digest the extracellular part of E-cadherin [25, 86, 87] and that overproduction of two *htrA* gene copies in *H. pylori* caused a more pronounced breakdown of E-cadherin compared to the 26695 wt strain in infected Caco-2 cells [47]. As we observed certain strain-dependent differences in the efficiency of infection, we decided to check the levels of HtrA secreted by various *H. pylori* strains. Strains 26695 and G27 exported higher levels of HtrA, compared to N6, J99 and 7.13 (Fig. 9a). Casein digestion by extracellular *H. pylori* proteins has shown that proteolytic activity correlated with the amounts of HtrA secreted by the strains (Fig. 9b and c). These strains also showed significant differences in HtrA secretion compared to other secreted proteins, especially in the case of UreB (Fig. 8c-e). In addition, casein digestion was verified using zymography. For the supernatant samples it was more difficult to demonstrate proteolytic activity due to the small amounts of secreted HtrA by strain N6, as confirmed above (Fig. 10).

## Conclusions

Proteases of the HtrA family exhibit both proteolytic and chaperone activities. In this work, we have expanded the previously published results, now focusing on the chaperone activity of the HtrA_*Hp*_ protein, which has not been characterized before. Analyses of the recombinant protein have shown that HtrA_*Hp*_ possesses chaperone activity that inhibits the aggregation of lysozyme and is stable under various pH and temperature conditions. Using a previously constructed Δ*htrA* deletion knockout and protease-deficient HtrA point mutant in strain N6 [27, 28], we have now demonstrated the importance of chaperone activity under thermal, pH and osmotic stress conditions. Moreover, we have proven that the lack of *htrA* in *H. pylori* does not affect the expression levels of many other proteins (both cellular and secreted). In addition, we could demonstrate that *H. pylori* lacking the proteolytic activity of HtrA exhibit reduced the levels of secreted HtrA. We also compared the secretion levels of HtrA from different *H. pylori* strains and showed significant differences in the amount of secreted HtrA, correlating with different efficiencies in the digestion of the substrate β-casein. Moreover, we have demonstrated the significant effect of HtrA on E-cadherin during infection of human polarized epithelial cells.

## Supplementary information

**Additional file 1:** **Figure S1.** Schemes of the *htrA* locus in *H. pylori* and the flanking chromosomal regions. **Figure S2.** Survival of the stress-exposed *H. pylori* cells. **Figure S3.** Effects of oxidative stress on the growth of various *H. pylori* strains. **Figure S4.** Mutation of *htrA* has no effect on the secretion of various proteins by *H. pylori*.

## Data Availability

The datasets supporting the conclusions of this article are included within the article and its additional files.

## Abbreviations

CagA: Cytotoxicity-associated immunodominant antigen
*Cj*: *Campylobacter jejuni*
ClpB: Chaperone protein ClpB
DnaJ: Chaperone protein DnaJ
DnaK: chaperone protein DnaK
FITC: fluorescein isothiocyanate
GGT: Gamma-glutamyltransferase
GroEL: 60 kDa chaperonin/ Heat shock protein 60
Hp: *Helicobacter pylori*
HtrA: High- Temperature Requirement A
Ibp: small heat shock protein
Lon: Lon protease
NapA: Neutrophil-activating protein NapA
UreB: Urease subunit beta
VacA: Vacuolating cytotoxin autotransporter
