# Supplementary material for: Chaperone activity of serine protease HtrA of Helicobacter pylori as a crucial survival factor under stress conditions
Source: Cell Commun Signal. 2019 Dec 3;17:161. doi: 10.1186/s12964-019-0481-9 (PMC6892219; doi:10.1186/s12964-019-0481-9)
Supplement: Supplementary file 1 — Additional file 1: Figure S1. Schemes of the htrA locus in H. pylori and the flanking chromosomal regions. Figure S2. Survival of the stress-exposed H. pylori cells. Figure S3. Effects of oxidative stress on the growth of various H. pylori strains. Figure S4. Mutation of htrA has no effect on the secretion of various proteins by H. pylori. [file 12964_2019_481_MOESM1_ESM.docx]

**Chaperone activity of serine protease HtrA of *Helicobacter pylori* as a crucial survival factor under stress conditions**

Urszula Zarzecka^1, 2^, Aileen Harrer^1^, Anna Zawilak-Pawlik^3^, Joanna Skorko-Glonek^2,^, and Steffen Backert^1,*^

^1^ Division of Microbiology, Department of Biology, Friedrich-Alexander-University Erlangen-Nürnberg, Erlangen, Germany.

^2^ Department of General and Medical Biochemistry, Faculty of Biology, University of Gdańsk, Gdańsk, Poland.

^3^ Department of Microbiology, Hirszfeld Institute of Immunology and Experimental Therapy, Polish Academy of Sciences, Wroclaw, Poland

**Additional information**

**Bacterial strains**

For experiments, we used the following *H. pylori* strains: (1) N6wt, (2) N6 Δ*htrA*, complemented strains with (3) wt *htrA* (Δ*htrA*/*htrA*_N6_) and with (4) a proteolytically inactive variant of *htrA* (Δ*htrA*/*htrA*_N6_ S/A).

**Statistic**

All statistical analyzes were performed using the Bonferroni test.


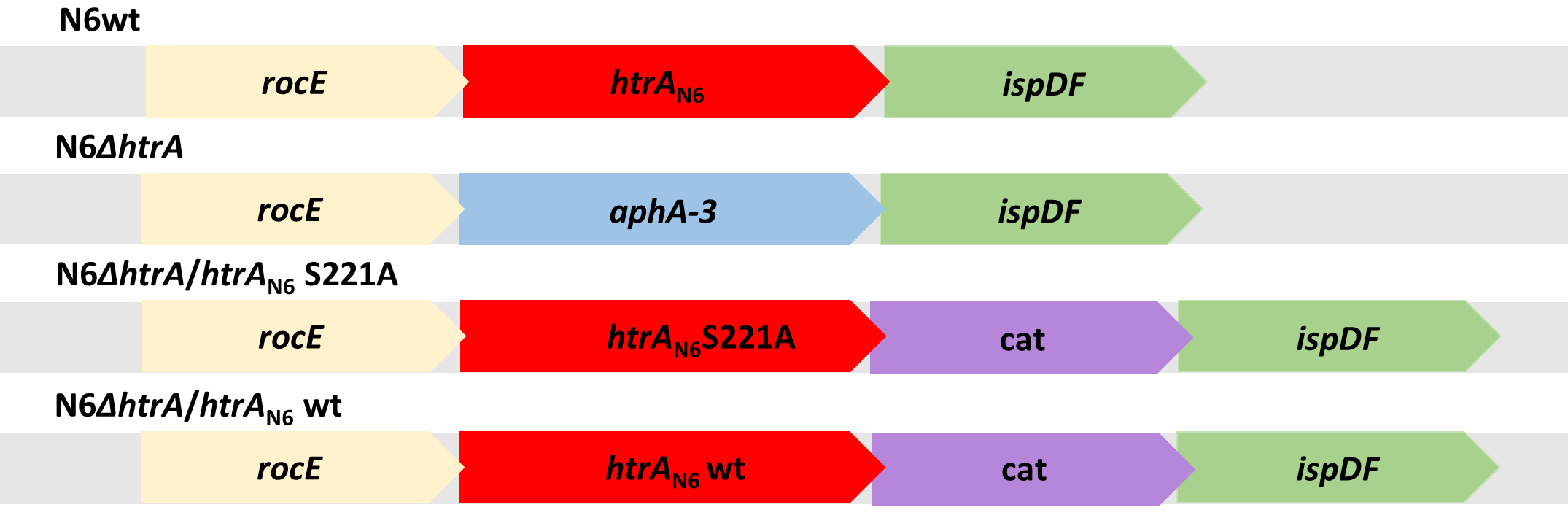


**Additional file 1: Figure S1.** Schemes of the *htrA* locus in *H. pylori* and the flanking chromosomal regions. The *htrA* gene locus comprises 3 indicated genes, *rocE*, *htrA* and *ispDF*, which are shown for the strains used in the present study*.*


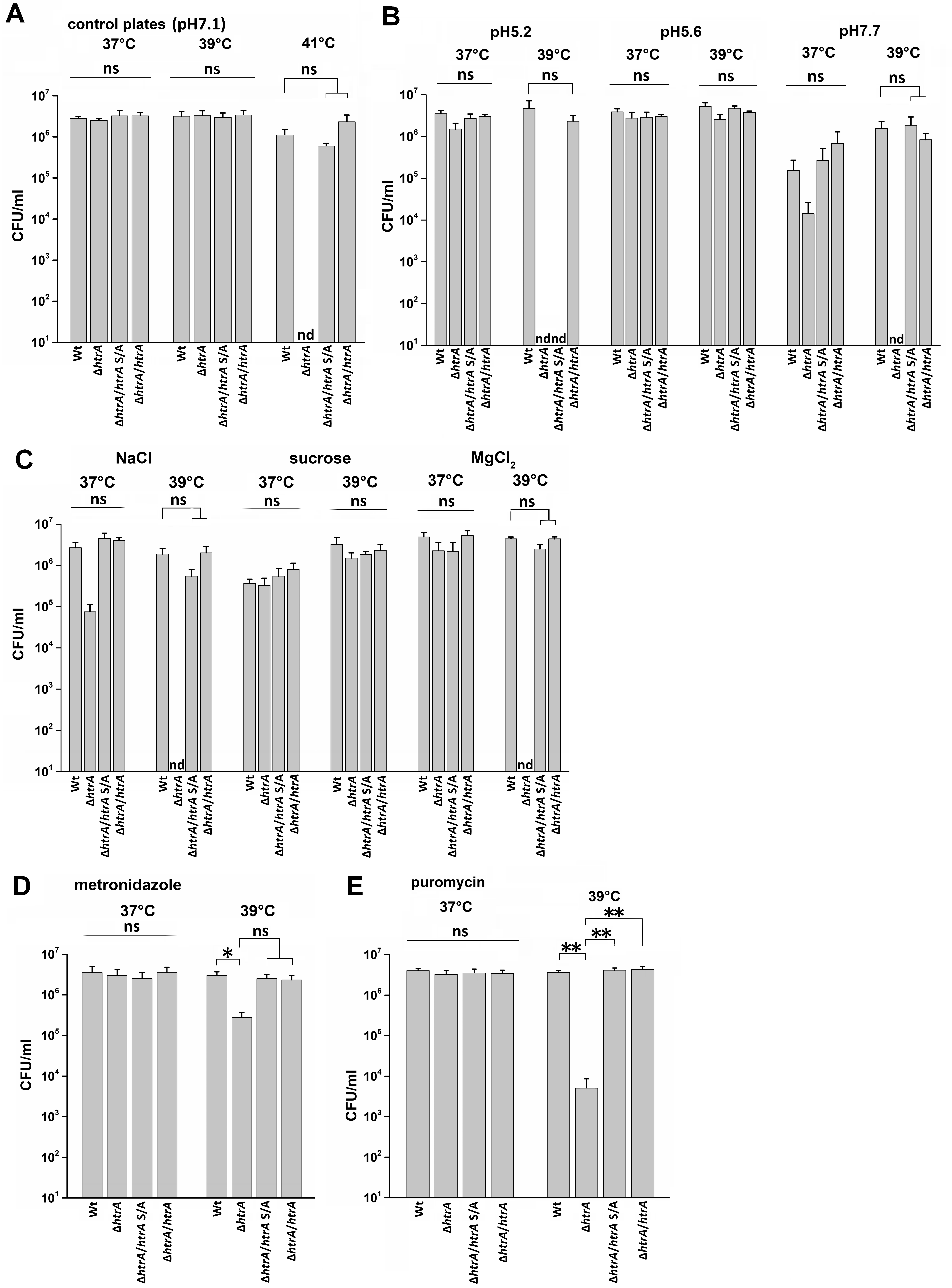


**Additional file 1: Figure S2.** Survival of the stress-exposed *H. pylori* cells. The *H. pylori* growth was tested under the indicated stress conditions as presented in Fig. 3a, 4a/b and 5a/b/c that correspond to panels **(a**), **(b),** **(c)**, **(d)** and **(e)** of this figure, respectively. The standard error of mean (SEM) was calculated using at least three repetitions. Statistical significance was defined by *p ≤ 0.05* (*). Nd - no single colonies detected; ns- no significant differences.


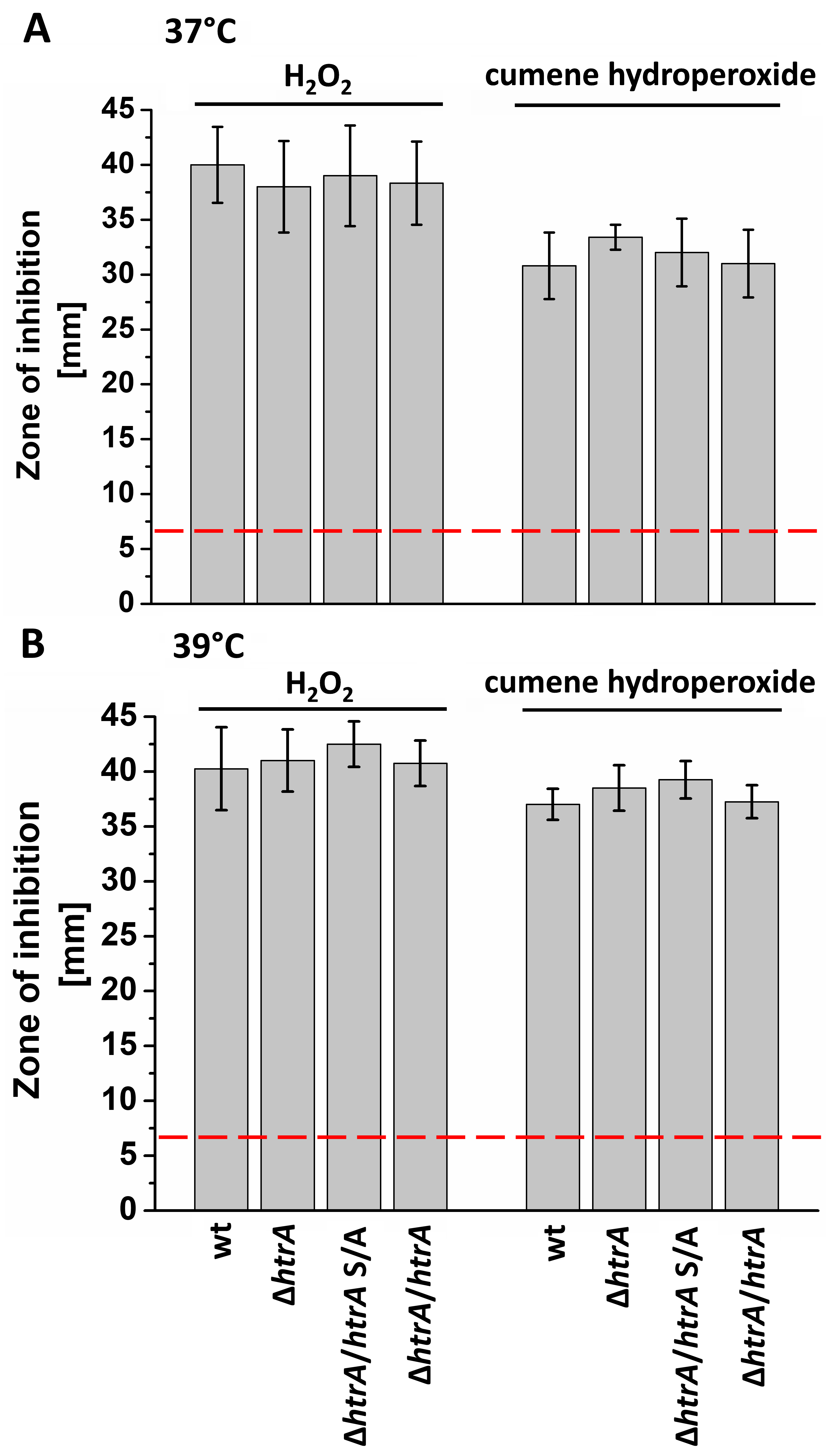


**Additional file 1: Figure S3.** Effects of oxidative stress on the growth of various *H. pylori* strains. The *H. pylori* isolates were cultured and challenged with H_2_O_2_ and cumene hydroperoxide as described in the Materials and Methods section. GC agar plates with the bacteria were incubated for 3 days at **(a)** 37 °C and **(b)** 39 °C. The experiments were performed at least three times. The red dotted line indicates the diameter of the filter disc. The statistical analysis showed no significant differences (p < 0.05) between the tested strains for all experimental conditions.


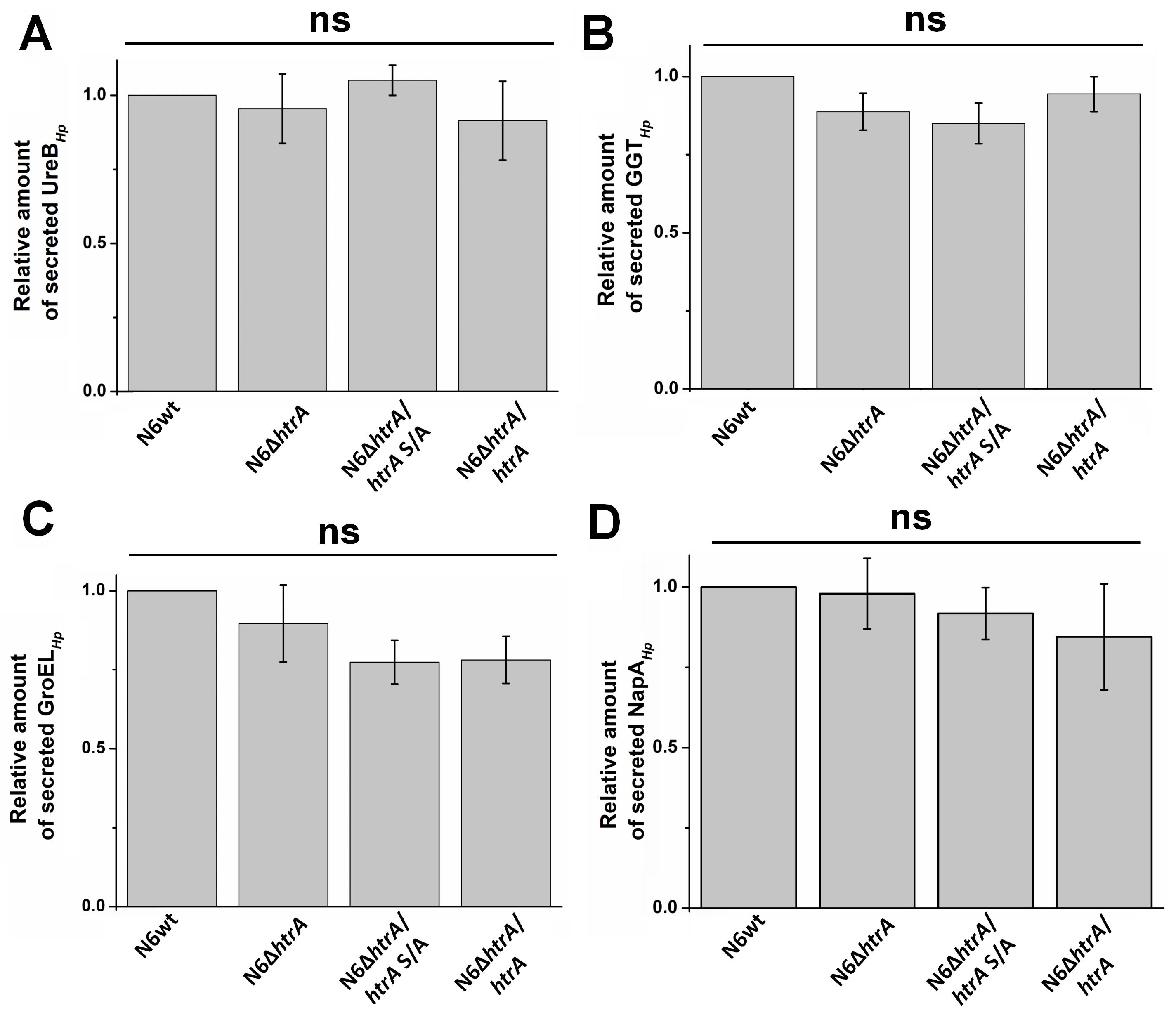


**Additional file 1: Figure S4.** Mutation of *htrA* has no effect on the secretion of various proteins by *H. pylori*. The following secreted proteins were tested: **(a)** UreB, **(b)** GGT, **(c)** GroEL and **(d)** NapA. Band intensities of secreted proteins were quantified densitometrically using 1Dscan Ex program and the relative amount of secreted protein is given. The standard error of mean (SEM) was calculated using at least three repetitions. ns means no significant differences for *p<0.05*.
